# Quality management: where is the evidence? Developing an indicator-based approach in Kenya

**DOI:** 10.1093/intqhc/mzw147

**Published:** 2016-12-09

**Authors:** Helen Prytherch, Maureen Nafula, Charles Kandie, Marc Brodowski, Irmgard Marx, Sandy Kubaj, Irene Omogi, Alexia Zurkuhlen, Claudia Herrler, Katja Goetz, Joachim Szecsenyi, Michael Marx

**Affiliations:** 1 evaplan at the University Hospital, Heidelberg, Germany; 2 Swiss Tropical and Public Health Institute, University of Basel, Basel 4002, Switzerland; 3 Institute of Health Policy, Management and Research (IHPMR), Nairobi, Kenya; 4 Head of Department of Standards and Regulatory Services, Ministry of Health, Nairobi, Kenya; 5 Institute for Applied Quality Improvement & Research in Health Care (AQUA), Göttingen, Germany; 6 Deutsche Gesellschaft für Internationale Zusammenarbeit (GIZ) GmbH, Health Programme, Kenya; 7 Institute of Family Medicine, University Hospital Schleswig-Holstein, Luebeck, Germany; 8 Department of General Medicine and Health Service Research, University of Heidelberg, Heidelberg, Germany; 9 Institute of Public Health, University of Heidelberg, Heidelberg, Germany

**Keywords:** quality improvement, quality indicators, health care, health services, maternal and child health, developing countries

## Abstract

**Introduction:**

The 2030 Sustainable Development Agenda emphasizes the importance of quality of care in the drive to achieve universal health coverage. Despite recent progress, challenges in service delivery, efficiency and resource utilization in the health sector remain.

**Objective:**

The Ministry of Health Department of Standards and Regulations sought to operationalize the Kenya Quality Assurance Model for Health. To this end, the European Practice Assessment (EPA) was adapted to the area of Reproductive and Maternal and Neonatal Health.

**Methods:**

The adaptation process made use of a ten step-modified RAND Corporation/University of California Los Angeles (UCLA) Appropriateness Method. The steps included a scoping workshop, definition of five critical domains of quality in the Kenyan context (‘People, Management, Clinical Care, Quality & Safety, Interface between inpatients and outpatients care’), a review of policy documents, management and clinical guidelines, grey and scientific literature to identify indicators in use in the Kenyan health system and an expert panel process to rate their feasibility and validity.

**Results:**

The resulting 278 indicators, clustered across the five domains, were broken-down into 29 dimensions and assigned measure specifications. A set of data collection tools were developed to furnish the indicators and piloted at two health facilities. They were subsequently finalized for use in 30 health facilities in 3 counties.

**Conclusions:**

The integrative and indicator-based aspects of the EPA process could be readily adapted to facilitate the operationalization of a practical quality assurance approach in Kenya.

## Introduction

Concerns about the quality of health care have risen high on the international agenda in recent years, as countries strive to strengthen their health systems and deliver universal health coverage. Evidence suggests that even where health care utilization rates increase in many low and middle income countries (LMIC) [1, 2], this increase has not been matched by a corresponding decline in mortality and morbidity rates [3] and the quality of care provided in both the public and private sectors of LMIC is considered a likely explanation of this discrepancy [4].

Despite notable efforts on the part of Government, development partners and other actors, the health sector in Kenya continued to be plagued by challenges in service delivery, efficiency and resource utilization. Beyond the problems of lacking infrastructure and shortages of equipment, drugs and staff, there are also known deficiencies in the quality of care. These are especially pronounced in the areas of Maternal and Neonatal Care, family planning and in the provision of services for the survivors of Sexual and Gender-based violence [5].

As a result, the maternal mortality rate remains unacceptably high at 360 per 100 000 live births [6]. Health-facility data indicated that 73% of pregnant women in Kenya attended at least one ante-natal care (ANC) visit in 2010, but survey data indicate many fewer—as low as 47%—accessed at least four visits, as recommended by WHO. Less than half of pregnant women (44%) delivered at a health facility in 2008–2009 [7]. Even these facility-based deliveries often take place under insufficient professional monitoring [8]. Important guidelines are either not available at facility level, or are not used [9]. Contraceptive prevalence is also low, with less than half of married women in Kenya (46%) using any method in 2008–2009 and stock-outs of contraceptives are common [5].

## Local problem

In 2009, the Kenya Quality Assurance Model for Health (KQMH) was established. A 2011 review of the KQMH found it to be suitably comprehensive in its scope but noted that only a minority of facilities had received any orientation on how it should be used and even in facilities where such training had taken place little implementation had been achieved [10]. With the ongoing roll out of a national health insurance scheme, demand for quality health services increased and the Kenyan Ministry of Health's Department of Standards and Regulatory Services (DSRS) sought to operationalize the KQMH and make it the point of reference for all facilities working to improve the quality of their services.

With support of the Deutsche Gesellschaft für internationale Zusammenarbeit (GIZ), the European Practice Assessment (EPA) was adapted to fill this implementation gap in Kenya. EPA is implemented in >2000 health facilities in Germany since 2003 as well as in six other European countries [11–14] with positive effect and is a recognized indicator-based approach [15].

## Methods

In collaboration with the Ministry of Health and DRSR a ten step-modified RAND Corporation/University of California Los Angeles (UCLA) Appropriateness Method process was used to develop the quality measures and adapt the EPA. The process is laid out in Table 1 and included a scoping workshop with quality improvement actors; the collation of health sector strategy, policy and planning documents, management and clinical guidelines, Standard Operating Procedures, grey and scientific literature; a two rounds panel process; the design of measure specifications; development of data collection tools, and piloting of the approach. Ethical clearance was obtained from the Institutional Research Ethics Committee (IREC) at Moi University, Kenya.

**Table 1:** Ten step-modified RAND/UCLA Appropriateness Method

Phase	Step
Planning	1. Scoping workshop
	– Exchange on different approaches to quality improvement in Kenya
	– Discussion to agree 5 possible domains of quality in the Kenyan context
	– Collation of existing national guidelines, documents, etc.
	2. Literature review
	– Review of literature and international guidelines
	– Paired review of resulting list of indicators to exclude duplicates/unsuitable indicators
	3. Organization of the assessment pannel
	– Identification of fifteen multidisicplinary experts
	4. Preparation of candidate indicators for the first-panel workshop
	– Definition of the indicator (numerator, denominator)
	– Inclusion and exclusion criteria
	– Sources
	– Clustering into five proposed domains
Rating	5. First-panel workshop
	– Overview of the development process
	– Provision of indicator templates
	– Assessment of the indicators according to SMART criteria
	– Identification of areas where indicators were missing
	– Validation of the proposed clustering into five domains
	6. Second-panel workshop
	– Provision of indicator templates for the updated register
	– Confirmation of the validity and feasibility of the indicators
Operationalizing	7. Specification of measures
	– Unit of analysis (patient, facility, provider, etc.)
	– Data sources (administrative data, medical records, survey, etc.)
	– Data collection procedures
	– Feedback strategies
Approval	8. Approval by Department of Standards and Regulatory Services
Piloting	9. Feasibility test
	10. Field testing

At the scoping workshop it was agreed that the Kenyan version of EPA, the Integrated Quality Management System (IQMS) would focus on the following five domains: ‘People (staff and patients), Management, Clinical Care, Quality & Safety and the Interface between inpatients and outpatients care’. Including the interface was considered crucial as most of the previous quality improvement efforts in the country had focused upon inpatients care, whilst in Kenya it is known that the majority of patient contacts occur *via* outpatients [16]. Moreover, it meant that an important emphasis was paid to the quality of the referral system—a notorious weak link in the provision of district health services [8] as well as to the relationship between facilities and communities.

### Structured search for indicators

The search comprised two steps. Firstly, health sector strategy, policy and planning documents, management and clinical guidelines, Standard Operating Procedures, and relevant grey literature were collated. Secondly, searches of the Pubmed and Direct Science databases were run using the terms ‘Quality of care’ and ‘Kenya’, with first abstracts and then full texts consulted. The resulting yield of documents were all reviewed for indicators already existing in areas related to the main domains; reproductive care, maternal and neonatal care, referral between facilities, services for survivors of gender-based violence, the interaction between facilities and communities, and general quality management strengthening. Relevant indicators were found in 40 of the documents as explained in [17]. At this stage, 456 possible indicators were identified.

**Figure 1:**
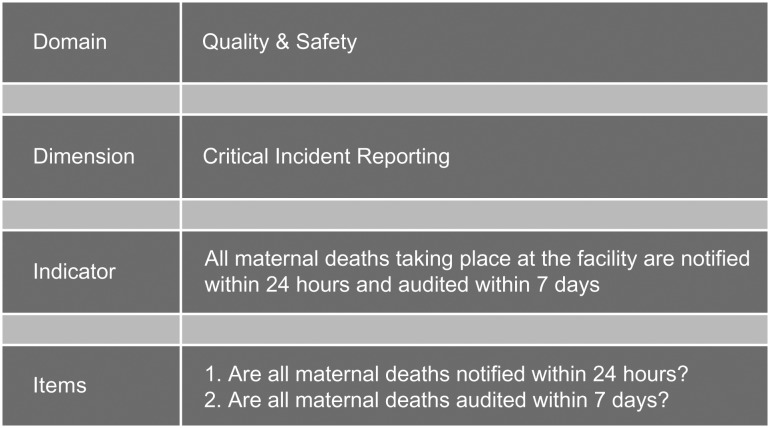
Overview of the different levels of the IQMS.

### Preparing candidate indicators for expert panel rating

A paired review of the indicators showed that there were often duplications or strong similarities between indicators. At this stage, duplicate indicators were deleted (57), and similar indicators from different sources combined, resulting in the removal of (34) indicators. In addition, indicators that facilities could not influence themselves, or were difficult to measure, for example not being suitable for repeated measurement overtime but rather one off recording were removed (45). The indicators were assigned to the five proposed domains, and clustered into possible dimensions so that these could also be reviewed by the expert panel. Only very few indicators for patient and staff satisfaction were identified from documents available within the Kenyan health system or scientific literature relating to Kenya. Internationally validated indicators were therefore referred to for these areas, and 22 indicators for patient satisfaction (3 indicators from EPA [18] and 19 from [19]) added, together with 22 indicators for staff satisfaction from EPA [19]. These changes resulted in a register of 364 candidate indicators.

**Table 2:** IQMS domains and dimensions

Domain	Dimension
Clinical care	Antenatal care
	Delivery and newborn care
	Postnatal care
	Family planning
	Survivors of gender-based violence
Interface inpatients/outpatients	Community
	General
	Referral
Management	Leadership and governance
	Drugs
	Supplies
	Maintenance
	Financial
	Data
	Equipment
	Amenities
	Transport
	Waiting times
People	Patient satisfaction
	Staff satisfaction
	Staff support
	Staff appraisal
	Staff general
Quality and safety	Critical incident reporting
	Emergency management
	General
	Guidelines, etc.
	Infection control
	Laboratory

Next, the indicators were prepared in a format showing their original formulation, their level of application—hospital or health facility (as applicable), the source document and possible questions or measurement prescriptions (items) that could be used to acquire the data needed for each indicator were prepared. An example of the items developed for one indicator is provided in Fig. 1.

**Figure 2:**
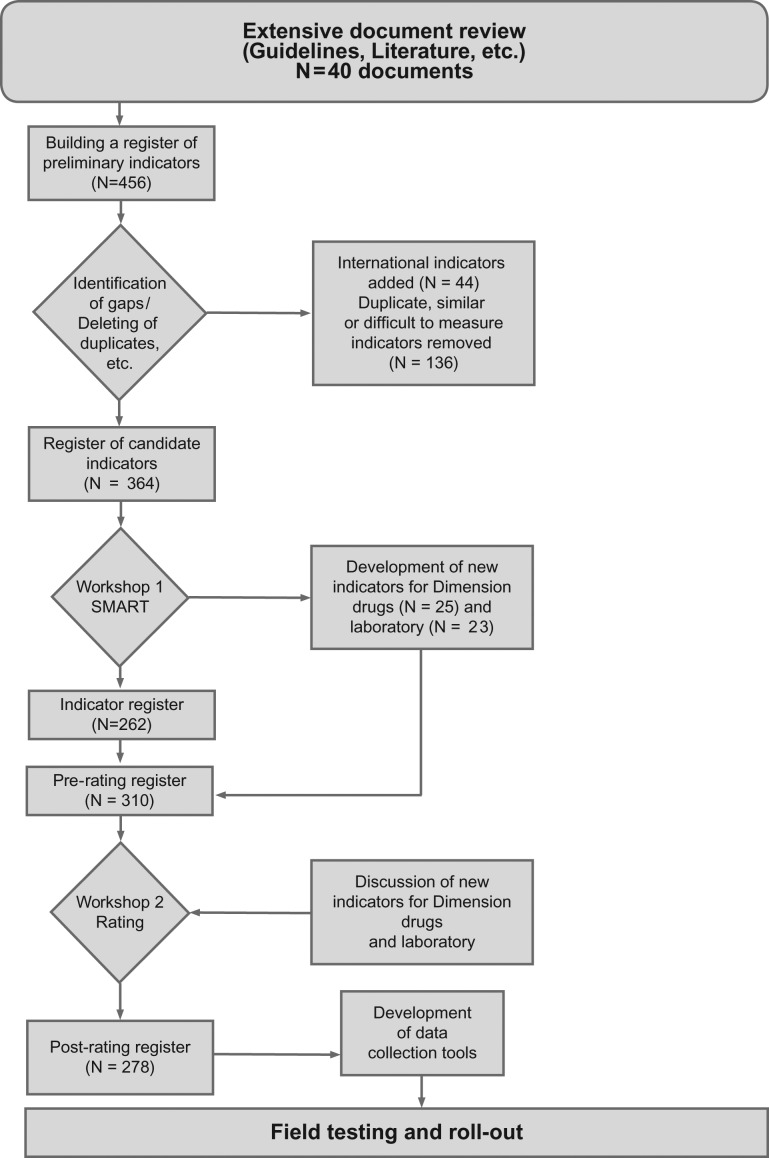
Overview of the process of establishing the indicators for use in the IQMS.

### Expert panel assessment: SMART workshop

A multidisciplinary panel of fifteen experts was invited to rate the indicators. Applicants were selected according to pre-defined criteria covering the clinical areas of interest at different levels of the health system, as well as from a policy and research perspective. The final selection included gynaecologists from district hospitals, nurses/midwives experienced in the provision of maternal, neonatal and child health services, as well as in caring for survivors of gender-based violence, a health-facility in-charge, a hospital manager, an academic familiar with Quality Management, representatives from the Division of Reproductive Health from province and district level, from the DSRS and the faith-based sector.

At the first workshop, the panel members reviewed the indicators, identified those of greatest relevance and assessed them according to the Specific, Measurable, Achievable, Relevant and Time-bound (SMART) criteria. In the case of the patient and staff indicators, the panel's function was to review and endorse their applicability in the Kenyan context.

Preference was given to indicators for which data were already being collected, e.g. in family planning, antenatal care and delivery registers and on post rape care forms to avoid generating additional workload at facility level, and to make the process of routine data collection more meaningful for the quality improvement effort. Upon the panel's advice, further expert opinion was sought to develop additional indicators on drug and laboratory services as this was found to be a gap and led to the development of 25 and 23 new indicators, which were added to the register in advance of the rating. After these steps were completed, there was an updated register of 310 indicators.

### Verification of indicators

In a next step, all the indicators in the updated register were reviewed to ensure they could be precisely measured as this reduces the risk of bias related to variation of results [18]. For example, if an indicator is assessed by use of a scale then the items/questions all needed to have clearly defined cutoff points. The formulation of the indicators also had to be checked to align with positive target achievement. High achievements needed to equate with a good result when compared with the rest of the indicator set.

### Rating workshop

The 310 indicators in the revised register underwent a rating process at the rating workshop. Analyses were based on the RAND/UCLA Appropriateness Method [20]. Indicators were rated for validity and feasibility. Indicators with a median higher 6 (≥7) on a 9-stepped ordinal scale and without a disagreement where considered face valid. Disagreement was defined as 30% or more of ratings in both the 1–3 tertile and the 7–9 tertile. All indicators with a median lower seven or a disagreement were considered invalid.

The experts then checked the weighting of the rate indicators between the five proposed domains. It was confirmed that all the twelve focus areas of KQMH were addressed by the five domains and twenty-nine dimensions of IQMS (See Table 2). In Table 3 the indicators from one dimension are illustratively shown. No further changes were made and the resulting parsimonious register of 278 indicators was used for all the next steps (Figure 2)

## Results

At the end of Step 9 in the RAND/UCLA Appropriateness Method, a parsimonious register of 278 indicators existed. To furnish these indicators, data collection tools were developed which included surveys for patients and staff, a self-assessment, facilitator assessment and a manager interview guide.

The ‘patient survey’ used a likert scale ranging from 1 (poor) to 5 (excellent), with an additional non-applicable/not relevant box. It required patients to confirm that they have used the facility at least twice before the current visit and to indicate the kind of services used at the current visit. The survey included questions on staff attitudes, how far patients felt listened to, received rapid relief of symptoms, encountered availability of drugs, clarification of instructions and privacy/confidentiality. Yes or no questions on whether fee schedules were prominently displayed, fees were paid, receipts issued or exemptions observed as applicable were also included.

The ‘staff survey’ also made use of a five-point likert scale, with an additional non-applicable/not relevant box. Staff were asked to agree or disagree with statements on whether supervision was provided in a supportive manner, team responsibilities were well defined, there is a good working atmosphere in the team, staff can voice their opinions without fear of reprisals, there was clear communication from management, equipment is available in working order, etc. Moreover, respondents are asked to express satisfaction with the working conditions (responsibility, remuneration, scope to use abilities, hours worked, possibilities for career development, etc.), as well as with relations with the community. Yes or no questions on whether the staff member had a signed contract, written job description and had undergone an annual appraisal in the last 12 months were also included. Some aspects of the staff survey were already published [21].

The ‘facility self-assessment’ comprised the auto-evaluative element of the IQMS. In advance of the assessment by the external facilitator, this is sent to the facility manager for completion. It draws heavily on readily available registers including the ANC register, family planning register, stock cards, maternity register, HMIS and laboratory records and focuses on acquiring quantitative data to furnish the indicators, e.g. total number of patients for different services and the proportion of them receiving various aspects of ANC, delivery and postnatal care. It also explored support provided to staff, staff appraisal processes, infection control, supply chain issues and aspects of financial management.

The ‘facilitator assessment’ maps closely to the self-assessment but also picks up aspects raised in the patient and staff surveys for triangulation purposes. As the facilitator already has the results of those two surveys when they visit the facility for the first time the assessment provides them with the opportunity to explore aspects that these may have raised. This assessment also has an element of verification, for example, requiring the assessor to look at random records to see how well they have been kept, and to establish stock levels and availability of functioning equipment on the actual day of the assessment.

Finally, the facilitator conducts an ‘interview with the facility manager’ using a pre-defined interview guide. This is designed to explore the synergies between the different areas, and to gain a deeper understanding of processes like efforts to motivate staff, or how to learn from critical incidents—where the discussion covered who does the analysis of such incidents, if there is formal structure for doing so, and an examination of some examples in the last quarter and what action was subsequently taken.

During the development of the tools, care was taken to make use of triangulation and to gather data for the sub-questions for each indicator from a variety of sources.

Table 3 shows the indicators and sources for one dimension in the clinical care domain. Table 4 illustrates how the indicators shown in Table 3 were reflected in illustrative items across the different data collection tools.

**Table 3:** Indicators from the domain clinical care, dimension delivery and newborn care with source

Percentage of macerated still births as proportion of total deliveries at facility in the last 12 months	International Indicator WHO
Percentage of pregnant women admitted into maternity with unknown HIV status that are counselled and tested for HIV during labour or after delivery during last month	Prevention of mother to child transmission (PMTCT) Guideline p. 90
Percentage of HIV-positive mothers admitted in maternity taking or reported to have taken the mother doses of preventive antiretrovial therapy (ARV) prophylaxis during last month	PMTCT Guideline p. 90
Percentage of infants born in facility receiving infant preventive ARV prophylaxis in maternity clinic during last month	PMTCT Guideline p. 90, Health Sector 2nd Ed. Indicators, SOP Manual (HIS), 2011 p. 58
Percentage of deliveries conducted by certified staff in the last 12 months	Health Sector Indicators and Standard Procedures – Popular Version p. 4, Health Sector 2nd Ed Indicators and SOP Manual (HIS), 2011 p. 5
Percentage of newborns with low birth weights (LBW) – (<2500 g)	Health Sector Indicators and Standard Procedures – Popular Version p. 4, Health Sector 2nd Ed Indicators and SOP Manual (HIS), 2011 p. 26
Percentage of maternal death reported at facility level in the last 12 months (calendar year)	Health Sector Indicators and Standard Procedures – Popular Version p. 6, Kenya Quality Assurance Model for Health Level 3 and 4 Check list, 2009 p. 28, Hospital reforms Supervision and Monitoring Tool 2010–2011 p. 8, DRH, M&E Framework, 2011–2012, p. 21
Percentage of perinatal deaths at the facility in the last 12 months (calendar year)	Kenya Quality Assurance Model for Health Level 3 and 4 Check list, 2009 p. 28
Percentage of fresh still births as proportion of total deliveries at facility in the last 12 months	International Indicator WHO
Percentage of births where correctly filled out partographs were used in the last month	Kenya National Reproductive Health Output Based Quality Improvement Accreditation and Assessment Tool, p. 14
The Facility has basic delivery equipment as per essential commodity list, the equipment is functional and maintained (scissors or blade, suction apparatus, disinfectant for cleaning perineum)	Kenya Service Provision Assessment (KSPA) 2010 p. 136; Norms and Standards
Percentage of perinatal deaths audited	New indicator, added by the panel at the first workshop

**Table 4:** Illustration of how the indicators in Table 3 were reflected in illustrative items across the different data collection tools

Staff survey	
There is good collaboration between my facility and traditional birth atendants	Likert scale 1 (strongly agree)-5 (strongly disagree)
Patient survey	
Questions (asked of maternity patients only)	
Were you ensured of privacy at the delivery?	Y/N
Did you get a hot drink after the delivery?	Y/N
Did you get anything to eat after the delivery?	Y/N
Did you receive sanitary pads after the delivery?	Y/N
Were you given warm bathing water after the delivery?	Y/N
Self-assessment	
Total number of macerated still births at the facility in the last 12 months	Provide number from maternity register
Total number of fresh still births at the facility in the last 12 months	Provide number from maternity register
Total number of deliveries in the facility in the last 12 months	Provide number from maternity register
Number of maternal deaths in the facility in the last 12 months	Provide number from maternity register
Total number of perinatal deaths	Provide number from maternity register
Total number of live births at the facility during the last 12 months	Provide number from maternity register
Facilitator checklist	Instruction
Total number of correctly filled out partographs in the last month	Look at the documentation of 10 randomly selected deliveries in the last month and enter number of times this was the case
Does the facility practice kangaroo mother care	Y/N
If yes, can staff members give a demonstration and explain when and how it should be used?	Y/N
In the equipment dimension	
The following basic equipment is available and functional: Weighing scale for newborns, scissors/blade, suction apparatus, disinfectant for cleaning perineum, drip stand, torches/portable lights	Y/N in each case. Yes only to be ticked if equipment is both available and functional on day of assessment
Is emergency support equipment for newborn care available and functional: external heat, oxygen, nasal gastric tube, laryngoscope, mucous extractor	Y/N in each case
* *In the amenities dimension	
Are the following basic amenities for service provision of maternity unit for level 2 and 3 available according to norms and standards: three examination coaches, three screens, two delivery beds, 10 delivery kits, one resusitation tray, oxygen, incubator, maternity beds, MWV kids, five stiching trays, CS kits, etc.	Y/N in each case
Does the labour ward provide privacy for clients?	Y/N in each case
* *In the infection control dimension	
Does the facility have a functional placental pit; is it lockable, is it concrete-lined with depth greater than 1 metre, is it inside the facility compound secured from unauthorized access?	Y/N in each case
* *In the drugs dimension	
Are the following available on day of assessment: antibiotics for newborn sepsis according to guidelines; ARVs or PMTCT according to guidelines; oxytocic according to guidelines, dextrose 5%; normal saline; ringer lactate; IV infusion set, etc.	Y/N in each case
* *Also aspects covered in the supplies, referral and community interface dimensions	
Manager interview	
Are the standard clinical guidelines available for active management of 3rd stage of labour?	Y/N
Is the implementation of this guideline in the daily routine work discussed with members of the clinical team?	Y/N
* *In the community dimension	
Do health promotion activities covering the importance of delivering at a facility take place at least quarterly?	Y/N
Also aspects covered in the referral and critical incident reporting dimensions	

### Piloting/field testing

Once the data collection tools had been developed, they were field tested at two facilities between January and February 2013. Staff were encouraged to fill out the staff survey whilst the project coordinator was still at the facility. For staff that were absent a collection box was left and arrangements made for collection at an agreed date. The patient survey was carried out orally in local languages. At least 100 responses per facility were sought from patients attending Antenatal Care, Postnatal, Family Planning and Maternity services. Once this number had been obtained, the process was considered complete. These surveys were complemented with the information received from the facility managers via their self-assessment.

A trained EPA facilitator with previous experience of working in sub-Saharan Africa supported the new quality facilitators to review the analysis of the data that had been collected from each facility in advance of the facility visits. This enabled them to start to generate a picture of the facility and where problems in need of greater examination might lie.

The process of the facility visits took 3 days and included a visit of the target departments/services (e.g. maternity ward, outpatient services, laboratory, pharmacy, central sterilization, administration, archive for medical records, ambulance, radio, kitchen compound, pit, laundry and airing facilities) for the facilitator assessment. The facilitators were accompanied by the head nurse of each facility and the respective responsible person of the service. Next, the interview with the Hospital (or Health Centre) Management Team was carried out using the interview guide. Facilitators then prepared the results and fed them back firstly to management and secondly to the rest of the team. Directly after the feedback sessions, the staff were supported to prepare a list of goals and priority areas and a plan of action for improvement including quick wins.

### Exchange on the field testing

A feedback workshop involving managers and staff was arranged for the two initial pilot facilities. The self-evaluative nature of the approach was well received. In particular, the process was praised for making the importance of data for planning and prioritization more clearly apparent. As the approach rests strongly on the use of national indicators, this meant that the results could easily be fitted into national planning and monitoring and evaluation processes.

The approach was found to be highly practical and appreciated for enabling facilities to set realistic priorities and commence the improvement process quickly without any additional significant resources. Both facilities were already able to present concrete steps they had already taken to improve quality since the field testing with the facility assessment took place. One facility received poor feedback about the attitude of their staff from the patient survey and had since arranged a focus group with women attending ANC to explore and address this.

After the field testing process, the tools were finalized. This did not involve any changes to the indicators, but at the level of the items/sub-questions some refinement were made and some additional items added to make the measurement clearer. In addition, the sequencing was adjusted, for example to enable the facilitator to deal with all issues on a certain topic, or gather all information from a single registry before moving to the next aspect. IQMS is currently used in 30 health facilities in 3 counties.

## Conclusions

The EPA approach is highly integrative and takes pre-existing indicators from national systems as well as available QI initiatives as it starting point. With the exception of the 44 international indicators that were retained through the review and rating process, 234 of the 278 indicators used in the ISQM had previously existed in the Kenyan health system.

In addition to exploring clinical areas, the approach offers the possibility to illuminate health system bottlenecks like drug distribution systems and facility accounting issues. The data collected via the various tools are transferred into indicators and visually displayed for feedback to the facility team as an integral part of the facility visit process. This breathes life into the process of collecting data for indicators. Based upon the objective and precise measurement and presentation of detailed results, facility teams can be supported to set their own quality targets making best use of existing resources according to the principle of Pareto. Moreover, the approach offers the possibility for facilities to compare baseline results with those of subsequent assessment so they can chart their progress. It also allows for the results of a facility assessment to be benchmarked against the average result of all the participating facilities.

Adapting the EPA in Kenya did support the operationalization of the KQAM, in particular because it provided facility level health managers with a practical tool for responding to patient demands and optimizing quality in small, effective ways without having to first wait to mobilize extensive resources.
